# The Effectiveness of Rambutan (*Nephelium lappaceum* L.) Extract in Stabilization of Sunflower Oil under Accelerated Conditions

**DOI:** 10.3390/antiox3020371

**Published:** 2014-05-09

**Authors:** Winne Sia Chiaw Mei, Amin Ismail, Norhaizan Mohd. Esa, Gabriel Akyirem Akowuah, Ho Chun Wai, Yim Hip Seng

**Affiliations:** 1 Department of Food Science and Nutrition, Faculty of Applied Sciences, UCSI University, No.1, Jalan Menara Gading, UCSI Heights, Cheras, Kuala Lumpur 56000, Malaysia; E-Mails: agabriel@ucsiuniversity.edu.my (G.A.A.); cwho@ucsiuniversity.edu.my (H.C.W.); hsyim@ucsiuniversity.edu.my (Y.H.S.); 2 Department of Nutrition and Dietetics, Facutlty of Medicine and Health Sciences, Universiti Putra Malaysia, 43400 UPM Serdang, Selangor 43400, Malaysia; E-Mails: aminis@upm.edu.my (A.I.); nhaizan@upm.edu.my (N.M.E.)

**Keywords:** rambutan, oxidative stability, peroxide value, *p*-anisidine value, iodine value, TOTOX, free fatty acid

## Abstract

The oxidative properties of sunflower oil supplemented with rambutan extract, (crude extract and its fractionated fraction, SF II) in comparison with synthetic antioxidant were investigated. The supplemented sunflower oils were stored under accelerated conditions for 24 days at 60 °C. For every 6-day interval, the oxidative properties of the supplemented sunflower oil were evaluated based on the following tests, namely peroxide value, *p*-anisidine value, Thiobarbituric Acid Reactive Substances (TBARS) assay, iodine value and free fatty acids. The total oxidation (TOTOX) values were also calculated based on the peroxide values and *p*-anisidine values. Rambutan extract is a potential source of antioxidant. The oxidative activities of the extracts at all concentrations were significantly (*p* < 0.05) higher than the control. Generally, the partially fractionated fraction was more effective than the crude extract. With a 2-year storage period at ambient temperature, the fractionated fraction of the extract, SF II at 300 ppm, was observed to work more effectively than the synthetic antioxidant, *t*-Tocopherol, and it possessed a protective effect comparable with butylatedhydrioxynanisole (BHA). Therefore, rambutan extract could be used as a potential alternative source of antioxidant in the oil industry or other fat-based products to delay lipid oxidation.

## 1. Introduction

Vegetable oils such as corn oil, canola oil, sunflower oil, and olive oil are recommended as ideal cooking media. They are high in polyunsaturated fatty acids that have many beneficial effects on the human body especially in lowering cholesterol [1]. However, these fatty acids are unstable and susceptible to oxidation [2]. Lipid oxidation is the principal cause of food deterioration. It is induced when lipids are exposed to environmental factors such as air, light or high temperature [2]. It produces off-flavor compounds and causes an unpleasant taste of the oil. Eventually, it decreases the quality and shortens the shelf life of the oil [3]. It also reduces the nutritional quality of the oil and produces toxic compounds [4].

In order to delay lipid oxidation, synthetic antioxidants have been widely used to maintain the quality and extend the shelf life of oils. Butylated hydroxyanisole (BHA), butylated hydroxytoluene (BHT), and *tert-*butyl hydroquinone (THBQ) are among the examples of commonly used synthetic antioxidant [3,4]. Recent has literature revealed that synthetic antioxidant may possess potential health risks, which include cancer [5]. In order to overcome this challenge, food industries have started to search for alternative antioxidants originating from natural sources. This reason for this is that antioxidants from natural extracts are presumed to be safe as they are from plants.

Studies have been done to evaluate the effectiveness of the use of natural extracts in delaying lipid oxidation. Rosemary, garlic, kenaf seed, roselle seed, coffee bean, catnip, sage, thyme, potato peel and sugar beet pulp are among the examples of natural extracts that have been found to work effectively in preventing oil rancidity [3,6,7,8,9,10]. According to the literature, the antioxidant potentials of the natural extracts are mainly contributed to the phenolic compounds present in the extracts. Tropical fruits are well known for their high phenol content [11]. Up to date, only a few studies have described the efficiency of fruit extracts in stabilizing vegetable oil. In one study, Iqbal *et al*., employed pomegranate peel extract to stabilize sunflower oil under accelerated conditions. The results of the study suggest pomegranate peel to be an effective source of antioxidant to stabilize sunflower oil [12].

Rambutan is an attractive tropical fruit, which belongs to the family of Sapindaceae [13]. It is commonly consumed in Asian countries. The harvest of the rambutan fruits was estimated to be half a million tons every years. However, only the flesh of the fruit is consumable. As a result, large amounts of waste are produced from the peels and the seeds [13]. The peels of the rambutan were found to have a high content of phenolic compounds and to be a powerful source of antioxidants [14]. Hence, in this study, the peel of rambutan (*Nephelium lappaceum* L.) extract was added to sunflower oil to investigate the effectiveness of the extract in delaying oil rancidity under accelerated conditions. The oil rancidity was evaluated by measuring the primary and secondary oxidation products during the storage period. Apart from that, the effectiveness of the rambutan peel extract was compared with tocopherol and BHA, synthetic antioxidants.

## 2. Experimental Section

### 2.1. Materials and Reagents

Refined, bleached and deodorized (RBD) sunflower oil was obtained from MOI Foods Malaysia SDN BHD, Selangor Malaysia. Rambutan fruits were purchased from a Malaysia local market. All the chemicals and reagents used were of analytical grade purchased from Merck (Darmstadt, Germany). BHA and Tocopherol were purchased from Sigma Chemical Co (St. Louis, MO, USA). Water used was Millipore quality (Millipore, Billerica, MA, USA).

### 2.2. Extraction Process

The fruit samples were washed and air-dried, followed by drying in an oven at 40 °C for 24 h. All the dried samples were ground to fine powder with a grinder, after which they were vacuum packed into a nylon-linear low density polyethylene film before analysis. An extraction with 80% ethanol was carried out, and the mixture was shaken at 50 °C for 120 min using an orbital shaking incubator as optimized in a previous study. The residue was subjected to another round of extraction under similar conditions to obtain maximal yield of antioxidant activities. The extracts were combined and centrifuged, followed by filtration and concentration of the supernatant using a rotary evaporator at 45 °C. The concentrated extract was freeze-dried, wrapped with aluminum foil and kept at −20 °C. The crude extract as then partially fractionated using silica packed open column chromatography and using increasing polarity of solvent, ethyl acetate, chloroform and methanol (100:0:0 to 0:0:100 v/v) [15]. In total, 40 fractions were collected. All the fractions were spotted on a thin layer chromatography to compare the Rf values. Fractions with similar Rf values were pooled together. As a result, three sub-fractions were collected. Fraction I was the pooling fraction from the flushing of 100% chloroform to ethyl acetate:methanol (8.5:1.5); Fraction II was collected from flushing of ethyl actetate:methanol (8:2) to ethyl actetate:methanol (3.5:6.5); Fraction III was collected from flushing of ethyl actetate:methanol (3:7) to 100% methanol. In this study, Sub-fraction II (SF II) was selected because it was found to have the strongest antioxidant activity and the highest phenolic content compared to the other sub-fractions. 

#### 2.2.1. Sample Preparation

In this study, the oxidative stability of sunflower oil was monitored under accelerated storage conditions according to the methods of Iqbal and Bhanger with some modifications [6]. All the samples were placed in amber glass bottles and stored in an oven at the fixed temperature of 65 °C for 24 days, whereby each day of storage was equivalent to one month of storage at ambient temperature [16]. The crude extract, and SF II were prepared at three different concentrations, namely 100 ppm, 200 ppm and 300 ppm. They were weighed and dissolved in a small amount of ethanol to facilitate dispersion in sunflower oil [17]. Tocopherol and BHA were also employed at their legal limit, 200 ppm, as references. All the samples (200 mL) were placed in amber class bottles and mixed for 30 min in an ultrasonic water bath at 60 °C. Apart from that, a control sample without antioxidants was also prepared and underwent the same treatment. The oxidative analyses (peroxide value, *p*-anisidine, TBARS, free fatty acid and iodine value) were conducted for 24 days for every interval of 6 days.

#### 2.2.2. Peroxide Value (PV)

The peroxide value (PV) was conducted by referring to the AOAC method 965.33 described by Zhang *et al.* [18,19]. Sunflower oil samples of 5.00 g were dissolved in 30 mL acetic acid-chloroform solution (3:2, v/v). After that, 1 mL of saturated KI was added. The mixture was allowed to stand with occasional shaking for one minute and kept in the dark for 5 min. This step was followed by addition of 30 mL distilled water. The mixture was titrated against sodium thiosulfate (0.002 M) until the yellow color almost disappeared. Then, about 0.5mL of 1% starch solution was added. The titration continued until the blue color disappeared. A blank was also analyzed under the same conditions. The peroxide value was calculated according the Equation:

Peroxide value (PV) = [S × M × 1000]/sample weight (g)
(1)

where, S is the value of Na_2_S_2_O_3_ used (blank corrected); M is the molarity of Na_2_S_2_O_3_.

#### 2.2.3. Measurement of *p*-Anisidine Value (AV)

The *p*-anisidine value (AV) was determined according to AOCS method Cd 18–90 [20]. Sunflower oil samples (2 g) were dissolved in 25 mL of isooctane and the absorbance was recorded at 350 nm. After that, 5 mL of the above mixture were mixed with 1 mL of 0.25% *p*-anisidine in acetic acid (w/v) and allowed to stand for 10 min. Lastly, absorbance was read again at 250 nm. The AV values were calculated using the Equation:

A*n*V= 25 × [1.2*A*_s_ − *A*]/sample weight (g) (2)

where *A*_s_ is the absorbance of test solution after reaction with the *p*-anisidine reagent; *A*_b_ is the absorbance of the fat solution.

#### 2.2.4. Total Oxidation (TOTOX) Values

Total oxidation (TOTOX) values of the oil samples were determined based on the obtained PV and AV values [7]. They were calculated using the following Equation:

TOTOX = 2PV + AV
(3)

#### 2.2.5. Thiobarbituric Acid Reactive Substances (TBARS) Assay

The lipid oxidations of the samples were evaluated using the 2-thiobarbituric acid (TBA) method according to Yim *et al.* [21]. In total, 10 g of sample were mixed with 10 mL of TBA-trichloroacetic acid (TCA) solution. The TBA-TCA solution was prepared by mixing 15% of TCA (w/v) and 20 mM of TBA in distilled water. The mixture was incubated in a water bath (90 °C) for 15 min until the pink color developed. After that, the samples were cooled in an ice bath for approximately 10 min and centrifuged at 4500 rpm for 15 min. The blank solution was prepared under the same conditions using distilled water. The absorbance of the supernatant was measure at 531 nm against the blank solution. A standard curve of malondialdehyde (MDA) was prepared using 1,1,3,3-tetraethoxypropane (TEP), and TBARS values were expressed as mg of MDA per kg of sample.

#### 2.2.6. Analysis of Iodine Value (IV)

The iodine value (IV) in the oil samples was determined using the Wijs method, as described in the AOAC official method 993.20 [18]. In total, 0.2 g of sample was dissolved in 15 mL of cyclohexane-acetic acid (1:1, v/v) solvent. Then, the mixture 25 mL of Wijs solution (16.5 g ICl was dissolved in 1 L acetic acid) was added, and the solution was kept in the dark at room temperature for 1 h. Then, the mixture 20 mL of 15% KI solution and 150 mL of distilled water were added. The mixture was gradually titrated against a 0.1 M Na_2_S_2_O_3_ solution while continuously being vigorously shaken until the dark brown color disappeared. The blank was analyzed under the same conditions. The IV was expressed as grams of iodine absorbed per 100 g sample (g I2/100 g) and was calculated using the Equation (3) below:

[IV = ((*B* − *S*) × *M* × 12.69)/sample weight(g)] (4)

where, *B* is the tritration of blank (mL); *S* is the titration of test solution (mL); *M* is the molarity of Na_2_S_2_O_3_ (mol/L).

#### 2.2.7. Free Fatty Acids (FFA)

The free fatty acids were determined according to the direct titration method with slight modification [19]. Firstly, a neutralized solution was prepared using phenolphthalein as indicator and a sufficient amount of sodium hydroxide (0.1 M) added to absolute ethanol until a faint permanent pink formed. After that, 7.00 g of oil samples were added into the oil and titrated against sodium hydroxide under vigorous shaking until a faint permanent pink appeared and persisted for longer than one minute. The result was expressed as percentage of free fatty acids (% FFA), which was calculated based on the following Equation:

FFA = (*V* × *C* × 56.11)/*m* (5)

where *V* is the volume of sodium hydroxide used after added oil samples; *C* is the concentration of sodium hydroxide; *m* is the mass of sunflower oil (g).

### 2.3. High Performance Liquid Chromatography Analysis (HPLC)

High performance liquid chromatography (HPLC) analysis of the rambutan crude extract was performed using Waters Delta 600 with 600 controller and photodiode array detector (waters 996). A phenomenex-Luna (5 µm) column (4.6 mm × 250 mm) was used. The mobile phase consisting of 0.1% aqueous formic acid (Solvent A) and acetonitrile (solvent B). The rambutan crude extract was run using the following gradient: first, it was operated with 95% Solvent A and increased to 75% Solvent A in 12 min; from 12 min to 30 min, the system was maintained running with 75% Solvent A. After that, Solvent A was increased to 85% in 2 min time and maintained for another 8 min. At 30 min, Solvent A was increased to 95% over a period of 5 min. The HPLC analyses were run at 280 nm. The sample volume injection was 10 µL. All the mobile phases were filtered through a 0.45 µm nylon membrane filter. The potent antioxidant was identified by comparing the retention time and spiking the sample with known injected standards.

### 2.4. Statistical Analysis

The experiment was repeated three times on different occasions. All analyses were carried out in triplicate (*n* = 9) for each replicate, in which the results were recorded as mean ± standard deviation; a one-way ANOVA analysis and Tukey’s multiple comparison were carried out using SPSS version 21 (SPSS Inc., Chicago, IL, USA) to compare the mean values of each treatment. The confidence level of 95% (*p* < 0.05) was used to determine the significant levels.

## 3. Results and Discussion

### 3.1. Peroxide Value

Hydroperoxide is the primary oxidation product produced as a result of lipid oxidation. It may break down into nonvolatile and volatile secondary products, which decrease the quality of the oil. This is an indicator of the initial stage of oxidative changes [22]. The presence of hydroperoxide in the oil can be determined based on the oxidation of iodine ion with hydroperoxide. A saturated iodine solution added to the oil sample reacts with the produced hydroperoxide from the lipid oxidation and releases free iodine as the end product. The liberated iodine is then titrated against sodium thiosulphate. The titration value can be calculated and reported as peroxide value, milliequivalents of oxygen per kilogram of sample (meq/kg) [23].

**Figure 1:**
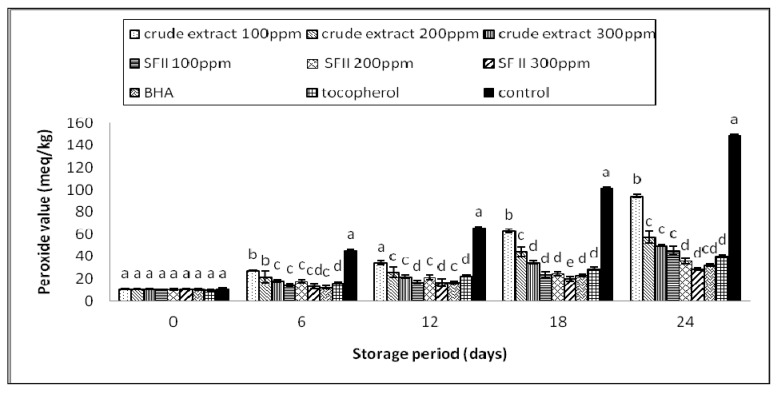
Peroxide value of the supplemented sunflower oil with rambutan extracts, butylatedhydrioxynanisole (BHA) and tocopherol under accelerated storage at 65 °C for 24 days.

In this study, the peroxide values of all the treated samples increased gradually with storage time as shown in Figure 1. The peroxide values of the samples ranged from 30.41 ± 3.64 to 148.69 ± 8.00 meq/kg after 24th day of storage. SF II at 300 ppm was reported to have the lowest peroxide value; this was followed by SF II 200 ppm; SF II 100 ppm, crude extract 300 ppm; crude extract 200 ppm and crude extract 100 ppm. It can be observed from the results that, as the sample concentration increased, the peroxide values decreased. Crude extract 300 ppm and the SF II 300 ppm were determined to have lower peroxide values than the crude extract 100 ppm and SF II 100 ppm. This revealed that the antioxidants were more effective in stabilizing the oil system at higher concentrations. These results were in accordance with the results reported by Iqbal *et al.* who tested the effectiveness of different concentrations of pomegranate peel extracts on the stabilization of sunflower oil [12].

The peroxide values for the synthetic antioxidants, tocopherol and BHA, were reported to be 40.66 ± 1.07 meq/kg and 30.41 ± 3.64 respectively, whereas it was 148.69 ± 8.00 meq/kg for the control after 24 days of storage. According to the results, the rambutan extract added to sunflower oil worked effectively. The peroxide value of the control was significantly (*p* < 0.05) higher than that of all the supplemented samples and synthetic antioxidants. This suggests that rambutan extracts were able to slow down the production of hydroperoxides, and hence delay lipid oxidation. For SF II at 200 ppm and 300 ppm, the peroxide values were significantly (*p* < 0.05) higher than for tocopherol, but not significantly (*p* < 0.05), different from those of BHA. Therefore, SF II with at a concentration of 300 ppm can be recommended as a potent source of antioxidants to stabilize oil systems. Apart from that, the PV values reported in this study are lower than those reported in sunflower oil with added pomegranate extract and garlic extract [6,12]. This suggests the superiority of the rambutan extract over other natural extracts in delaying primary oxidation in sunflower oil.

### 3.2. The p-Anisidine Value

The *p*-anisidine (AV) value is the measure of the secondary product produced when the hydroperoxide decomposes to carbonyl, aldehydes, and other compounds. This is the stage that leads to the rancid flavor of the oil [24]. The AV value can be obtained through color quantification, which is defined as the absorbance of a solution resulting from the reaction of 1 g of fat in isooctane solution (100 mL) with *p*-anisidine (0.25% in glacial acetic acid). The added anisidine reacts with the aldehyde and produces a yellow colored solution. The yellow color is measured at the absorbance value of 350 nm. A lower AV value indicates a better quality of oil [25].

**Figure 2:**
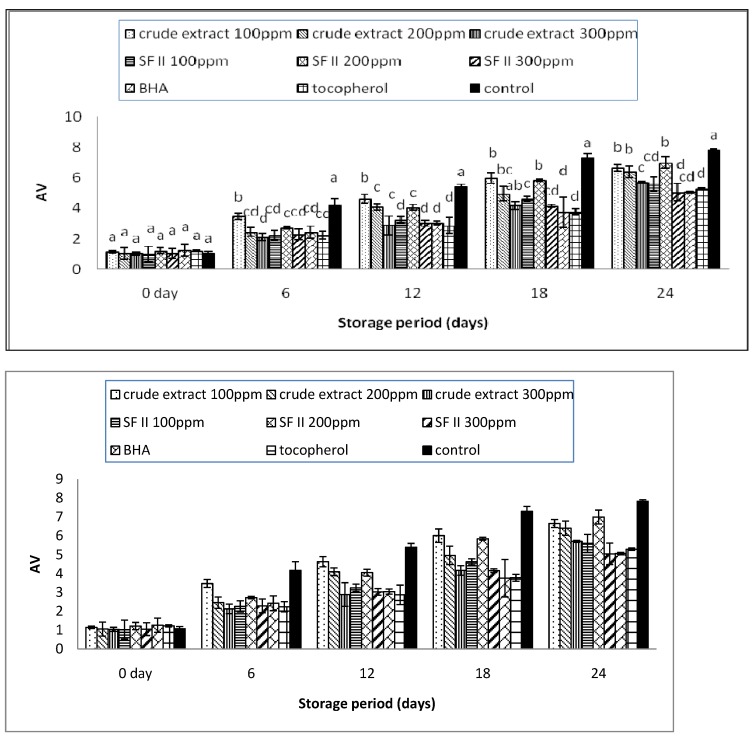
The *p*-anisidine (AV) value of the supplemented sunflower oil with rambutan extracts, BHA and Tocopherol under accelerated storage at 65 °C for 24 days.

The AV value of the treated samples showed a similar trend with the reported PV value, as shown in Figure 2. The AV values of the supplemented sunflower oil increased throughout the 24 days of storage. The crude extract at a concentration of 100 ppm was observed to be the least effective. From Day 6 onwards, the AV value of the crude extract 100 ppm was significantly (*p* < 0.05) higher than that of the other supplemented samples except for on the 24th day, where there was no significant (*p* < 0.05) difference to the crude extract at 200 ppm. This indicates that the crude extract at 200 ppm is only able to delay oil rancidity up to the 18th day, which is equivalent to 18 months of storage period under normal conditions. The active compounds present in the rambutan extract might deteriorate or decompose with storage time [21]. However, an increase in the sample concentration could extend the period, which was evidenced in the results of this study. The crude extract at 300 ppm reached a maximum value of 5.96 ± 0.05, which is lower than the reported value in the crude extract at 200 ppm. There was no significant (*p* < 0.05) difference to the synthetic antioxidant, tocopherol. An increase in the concentration of the sample was able to prolong the shelf-life of the oil.

After 24 days of storage, the AVs of the crude extract and sub-fractions were as follows: SF II 200 ppm > crude extract 100 ppm > crude extract 200 ppm > crude extract 300 ppm > SFII 100 ppm > SF II 300 ppm, with maximum values of 6.98 ± 0.37, 6.65 ± 0.21, 6.40 ± 0.38, 5.96 ± 0.05, 5.59 ± 0.48, 5.04 ± 0.57 after 24 days of storage. Overall, the sub-fraction was observed to be more effective than the crude extract. SF II showed significantly (*p* < 0.05) lower AV values than the other supplemented oil samples. Compared with the synthetic antioxidant, SF II at 300 ppm showed no significant (*p* < 0.05) differences to BHA, but had a significantly (*p* < 0.05) higher AV value than tocopherol. The protective effects of SF II at a concentration of 300 ppm were observed to be comparable with those of the synthetic antioxidants. It can be suggested as a potential source of antioxidants in the prevention of the production of secondary products in sunflower oil.

### 3.3. TOTOX Value

The total oxidation of the oil sample can be calculated based on the determined PV values and AV values. These values are reported as TOTOX value. These values reflect the initial and later stages of the oil oxidation. It measures the primary product, hydroperoxide, and its breakdown product, aldehyde. Therefore, it provides a better estimation of the progressive oxidative deterioration of the oil. The lower TOTOX value indicates a higher quality of the oil [18].

**Figure 3:**
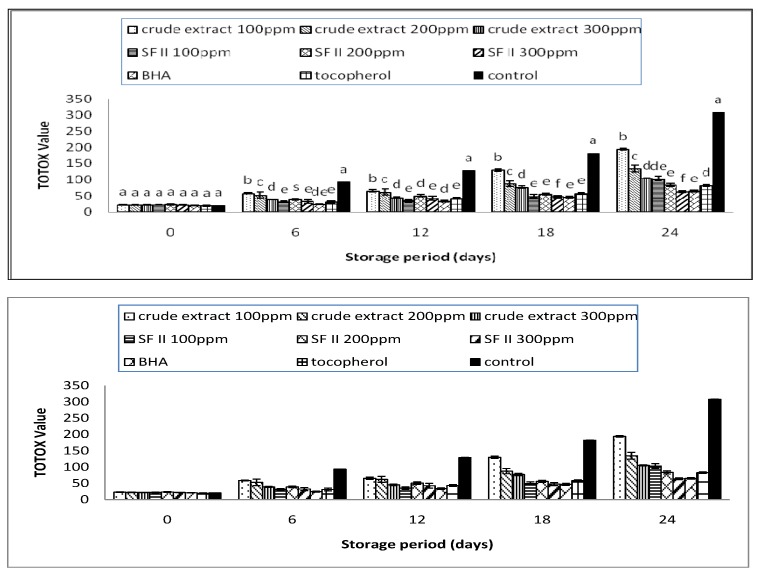
The TOTOX value of the supplemented sunflower oil with rambutan extracts, BHA and tocopherol under accelerated storage at 65 °C for 24 days.

The TOTOX values of the supplemented samples are as shown in Figure 3. All the supplemented samples showed positive effects in inhibiting oxidative rancidity. As compared to the control, all the supplemented samples were observed to have significantly (*p* < 0.05) lower TOTOX values. The TOTOX value of all the samples including synthetic antioxidants were as follows: control > crude extract 100 ppm > crude extract 200 ppm > crude extract 300 ppm > SF II 100 ppm > tocopherol > SF II 200 ppm > BHA > SF II 300 ppm with maximum values of 304.35 ± 5.69, 195.82 ± 2.66, 121.31 ± 0.33, 105.65 ± 1.58, 97.12 ± 7.47, 86.01 ± 2.49, 78.49 ± 4.90, 68.33 ± 2.08, 62.08 ± 2.62. The protective effects of the crude extract were observed to be lower than that of the sub-fraction SFII. SFII with a minimum concentration of 200 ppm was observed to have a comparative inhibitory effect to the synthetic antioxidant. After 24 days of storage, SF II at a concentration of 200 ppm was observed to be more effective than tocopherol; while at 300 ppm it showed significantly (*p* < 0.05) lower TOTOX values than tocopherol and BHA. For the crude extract, the protective effect was comparable with tocopherol only up to 12 days or equivalent to one year of storage at ambient temperature. In order to increase the protective effect of the crude extract, higher concentrations (up to 1000 ppm) can be implemented. It can be deduced from the TOTOX value that rambutan extract is able to delay the oxidative rancidity of sunflower oil for at least 2 years at ambient temperature.

### 3.4. Thiobarbituric Acid Reactive Substances (TBARS) Value

Malondialdehyde (MDA) is a component of the fatty acids with three or more double bonds. It is produced following the degradation of polyunsaturated fatty acids during lipid oxidation. The primary product, hydroperoxide reacts with oxygen to form MDA, which contributes to the off-flavor of the oil [19,24]. TBARS values have been widely used as the marker for oxidative stress [21]. The MDA levels in an oil sample can be determined through its reaction with thiobarbituric acid (TBA). In this test, MDA reacts with TBA to form the pink colored complex MA-TBA. The intensity of the pink color can be read spectrophotometrically at 535 nm. The TBA values are expressed as milligrams of MA equivalents per kilogram of sample [19].

**Figure 4:**
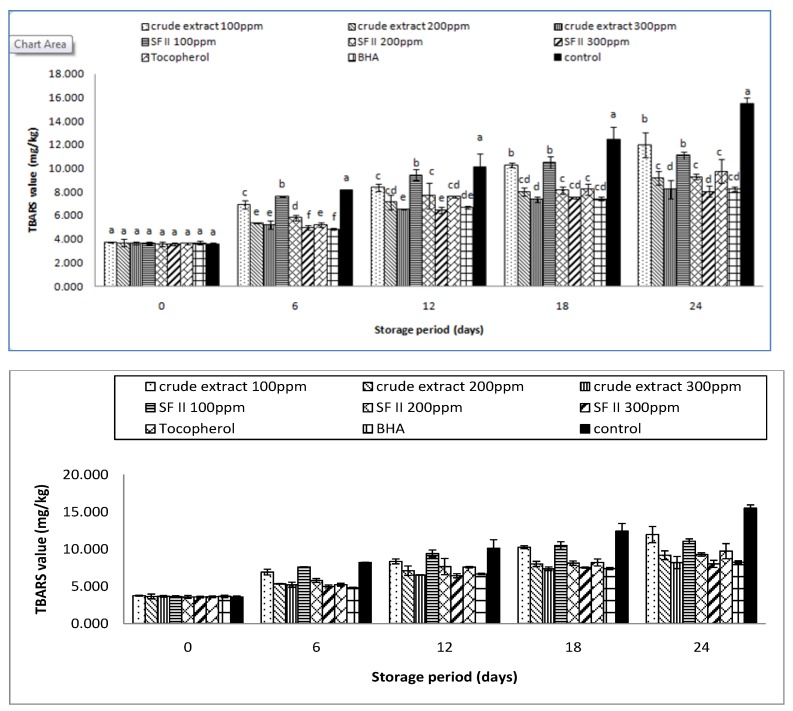
The thiobarbituric Acid Reactive Substances (TBARS) value of the supplemented sunflower oil with rambutan extracts, BHA and tocopherol under accelerated storage at 65 °C for 24 days.

The TBARS values of the supplemented samples stored for 24 days are as shown in Figure 4. The results show that rambutan extracts were able to inhibit the formation of TBARS at all concentrations. The TBARS values of all the supplemented samples and the control increased gradually from Day 0 to Day 24. As the sample concentration increased, the amounts of secondary products detected were lower. This result was in accordance with the obtained AV value. The secondary products were determined to be lower at higher concentrations. This result was also in accordance with the result obtained in the study of garlic extract and pomegranate peel added to sunflower oil, which reported to have stronger protective effects at higher concentrations. [6,12].

For both crude extract and SF II, the TBARS value decreased with increasing sample concentration. On the 24th day, the crude extract at 100 ppm showed a TBARS value of 11.98 mg/kg, the crude extract 200 ppm that of 9.19 ± 0.59 mg/kg and the crude extract at 300 ppm showed a TBARS value of 8.20 ± 0.81mg/kg. A similar trend could be observed in the sub-fraction At 100 ppm, a TBARS value of 11.17 ± 0.31 mg/kg was found; at 200 ppm a value of 9.21 ± 0.23 mg/kg and at 300 pm a value of 8.06 ± 0.48 mg/kg. The crude extract and SF II 300 ppm showed no significant (*p* < 0.05) effect after the 24th day of storage. The inhibitory effect of the sample at 300 ppm was significantly (*p* < 0.05) stronger than that of tocopherol with a TBARS value of 9.65 ± 1.01 mg/kg, and it was comparable with that of BHA (8.24 ± 0.19 mg/kg).

### 3.5. Iodine Value

Sunflower oil contains more than 70% polyunsaturated fatty acids. These polyunsaturated fatty acids are susceptible to lipid oxidation. During storage, the double bonds of these polyunsaturated fatty acids are attacked by free radicals, which results in the formation of conjugated bonds [7]. Thus, measuring the amount of unsaturated fatty acids present in the sunflower oil can be used as a reference to determine the freshness of the oil. The degree of “unsaturation” of the sunflower oil can be determined quantitatively by adding Iodine monochloride to the oil samples. The unsaturated fatty acids react with iodine monochloride and release free iodine. The free iodine can then react with sodium thiosulphate. The results are then calculated and expressed as Iodine value, g I_2_/100g [18].

**Figure 5:**
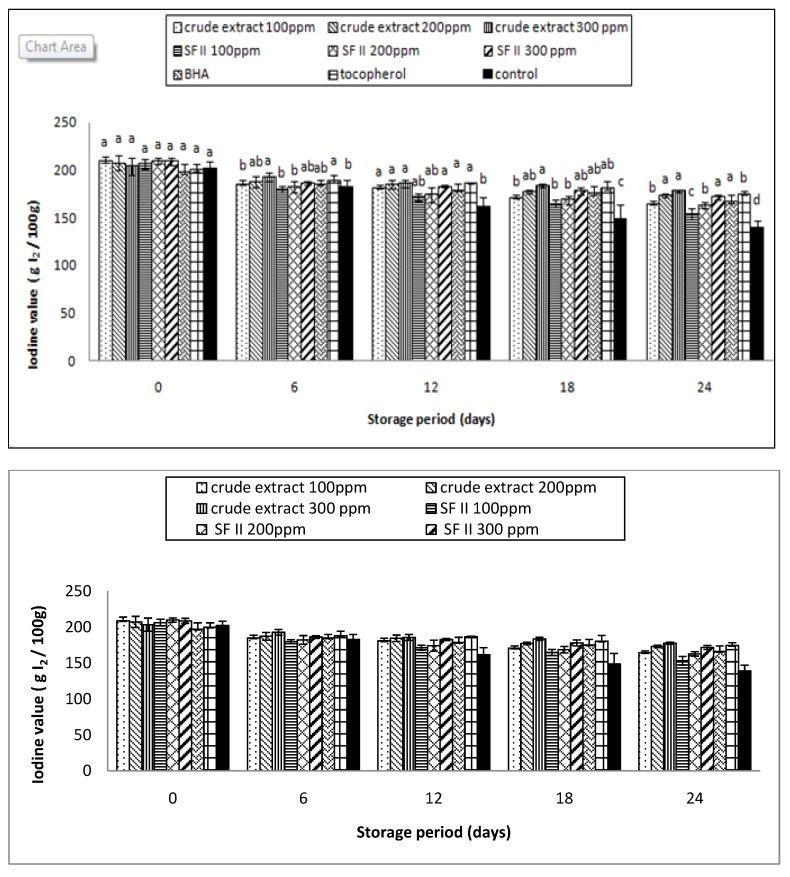
The Iodine value of the supplemented sunflower oil with rambutan extracts, BHA and tocopherol under accelerated storage at 65 °C for 24 days.

The IV value of the supplemented samples and the control are shown in Figure 5. A decreasing trend in the IV value can be observed in all samples. The IV results obtained are in agreement with the reported PV values. The PV values of all the samples increased due to the accumulation of hydroperoxides as a result of free radical attacking the unsaturated fat [7]. As a result of this free radical attack, the degree of unsaturation decreases and, hence, a decreasing trend of IV value can be observed with storage time. The IV values were in the range of 199.80 ± 6.15–209.81 ± 2.99 g I_2_/100 g on Day 0, and decreased to 139.31 ± 7.94–177.59 ± 1.43 g I_2_/100 g on Day 24.

Starting from Day 12, the IV value of the crude extract at 100 ppm was significantly (*p* < 0.05) lower than that of the other supplemented samples. On the 18th day, the crude extract at 300 ppm, the SF II 300 ppm, tocopherol, and BHA showed stronger inhibitory effects, while on 2the 4th day, no significant (*p* < 0.05) differences could be observed between the crude extract at 200 ppm, 300 ppm, SF II 300 ppm, BHA, and tocopherol. The IV values obtained in this study revealed that rambutan extract in a minimal concentration of 200 ppm could effectively delay the oxidation of the polyunsaturated fatty acids in sunflower oil. The protective effects of the rambutan extract are comparable with the synthetic antioxidant.

### 3.6. Free Fatty Acid

Free fatty acids (FFA) are produced due to the hydrolysis of triglycerides in the oil. The hydrolysis process can be promoted by the moisture content. It is considered one of the important indicators for oil rancidity [18].

**Figure 6:**
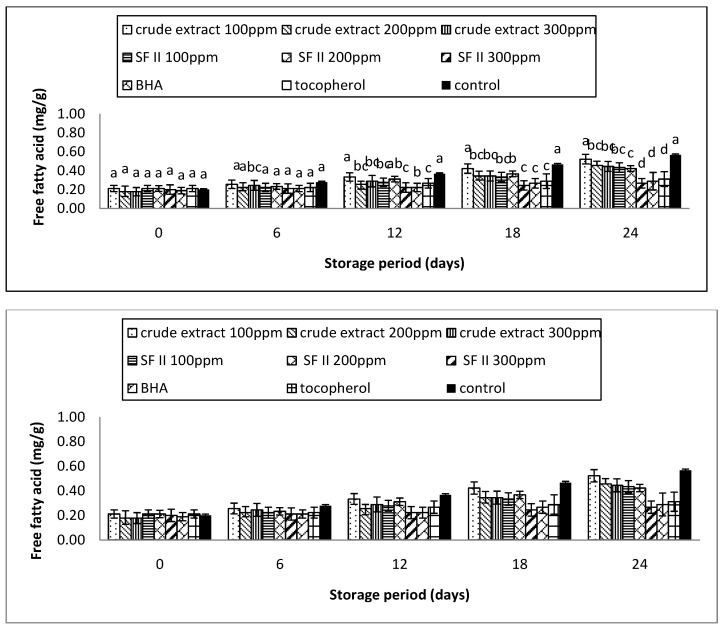
The free fatty acid of the supplemented sunflower oil with rambutan extracts, BHA and tocopherol under accelerated storage at 65 °C for 24 days.

The FFA content of the supplemented samples was as shown in Figure 6. After the 24th day of storage, the FFA of the all the treated samples were as follows: control (0.57 ± 0.05 mg/kg) > crude extract 100 ppm (0.52 ± 0.04mg/kg) > crude extract 200 ppm (0.46 ± 0.07 mg/kg) > crude extract 300 ppm (0.44 ± 0.05 mg/kg) > sub-fraction 100 ppm (0.43 ± 0.05 mg/kg) > sub-fraction 200 ppm (0.42 ± 0.04 mg/kg) > tocopherol (0.31 ± 0.08 mg/kg) > BHA (0.29 ± 0.09 mg/kg) > Sub-fraction II 300 ppm (0.27 ± 0.07 mg/kg). The FFA of the supplemented samples showed no significant (*p* < 0.05) difference for the first 6 days. An increasing trend in FFA values can be observed after 12 days of storage. Among the supplemented samples, the FFA of the Sub-fraction II at 300 ppm showed no significant (*p* < 0.05) differences compared to the synthetic antioxidants, tocopherol and BHA. The present results suggest that Sub-fraction II at 300 ppm is able to stabilize the oil system and that its protective effects are comparable with those of the synthetic antioxidants.

### 3.7. HPLC Analysis of Potent Antioxidant

**Figure 7:**
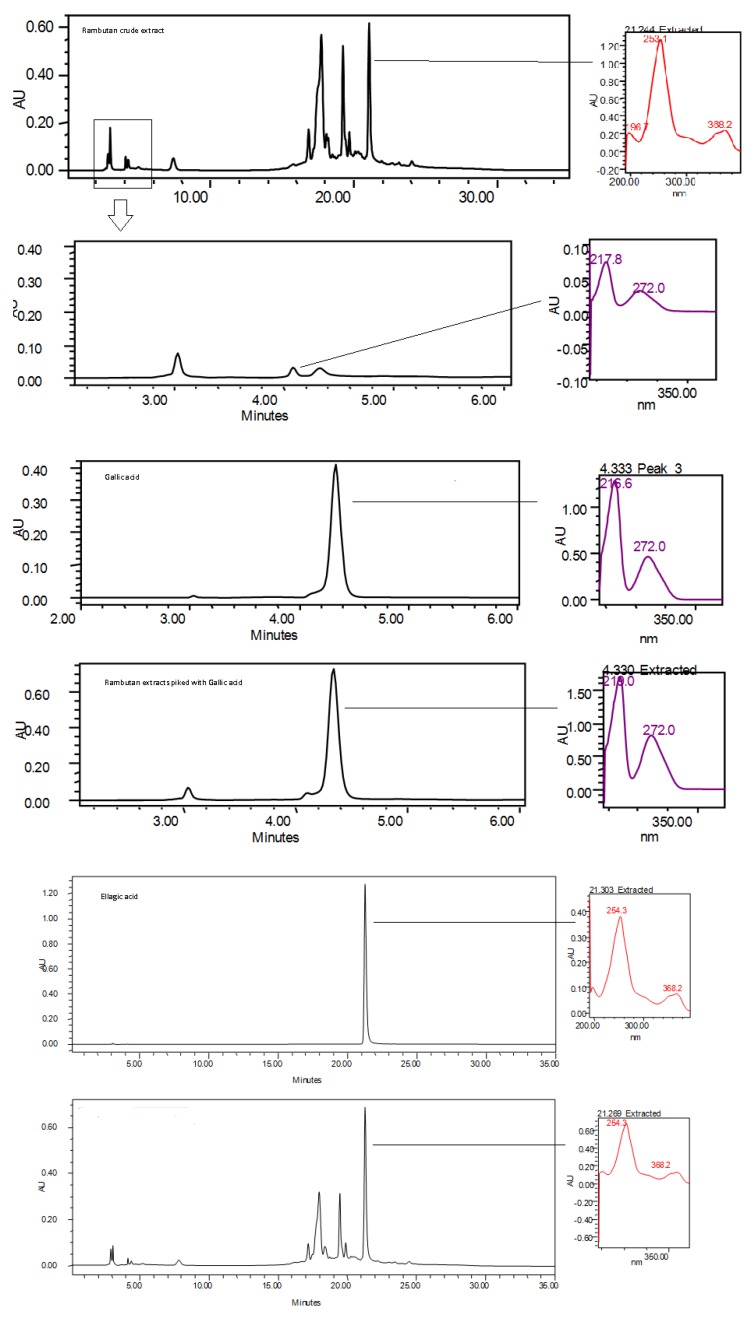
High performance liquid chromatography (HPLC) profiling of rambutan crude extract.

The HPLC analysis of the rambutan crude extract was as shown in Figure 7. The HPLC analysis detected the presence of gallic acid and ellagic acid in the rambutan crude extract as shown in Figure 7. Apart from that, there were a few unidentified peaks, which needed further analysis. The identified peaks were suspected to be either geraniin or corilagin. According to the literature, geraniin and corilagin are the common compounds found in rambutan [14] According to the results the HPLC analysis, gallic acid was observed to elute out at 4 min and ellagic acid at 21 min. The presence of the gallic acid and ellagic acid was confirmed by spiking the standard with the sample and by comparing the spectra of the samples as shown in Figure 7. This result was in accordance with the results reported by Thitilertdecha who also found the presence of ellagic acid in the extract of the rind of rambutan [13]. The presence of ellagic acid and gallic acid in the rambutan extract could contribute to the antioxidant effect of the extract, which delays oil oxidation.

## 4. Conclusions

This study shows that the extract of the peels of *rambutan* can be used as an alternative source of antioxidants for the stabilization of sunflower oil. It can be observed that the sub-fraction of the *rambutan* extract works more effectively than the crude extract. The SF II with a concentration of 300 ppm was observed to have a protective effect comparable with that of the synthetic antioxidant for 2 years of storage period at ambient temperature. For the crude extract at 300 ppm, comparable antioxidant activities with tocopherol were shown for only up to 1 year of storage. In order to improve the protective effect of the crude extract, higher concentrations of the sample can be incorporated into sunflower oil.
